# The prognostic relevance of FOXA1 and Nestin expression in breast cancer metastases: a retrospective study of 164 cases during a 10-year period (2004–2014)

**DOI:** 10.1186/s12885-019-5373-2

**Published:** 2019-02-28

**Authors:** Shahin De Lara, Jenny Nyqvist, Elisabeth Werner Rönnerman, Khalil Helou, Elisabeth Kenne Sarenmalm, Zakaria Einbeigi, Per Karlsson, Toshima Z. Parris, Anikó Kovács

**Affiliations:** 1 Department of Clinical Pathology, Sahlgrenska University Hospital, Gula stråket 8, SE-41345 Gothenburg, Sweden; 2 Department of Surgery, Skaraborgs Hospital, Lidköping and Sahlgrenska Academy at University of Gothenburg, Gothenburg, Sweden; 3 Department of Oncology, Institute of Clinical Sciences, Sahlgrenska Cancer Center, Sahlgrenska Academy at University of Gothenburg, Gothenburg, Sweden; 4 Center for Research and Development, Skaraborgs Hospital, Skövde, Sweden; 5 Department of Oncology, Sahlgrenska University Hospital, Gothenburg, Sweden

**Keywords:** FOXA1, Nestin, GATA3, Breast cancer metastases, Immunohistochemistry

## Abstract

**Background:**

Current prognostic markers cannot adequately predict the clinical outcome of breast cancer patients. Therefore, additional biomarkers need to be included in routine immune panels. FOXA1 was a significant predictor of favorable outcome in primary breast cancer, while Nestin expression is preferentially found in triple-negative tumors with increased rate of nodal metastases, and reduced survival. No studies have investigated the prognostic value of FOXA1 and Nestin expression in breast cancer metastases.

**Methods:**

Breast cancer metastases (*n* = 164) from various anatomical sites were retrospectively analyzed by immunohistochemistry for FOXA1, Nestin and GATA3 expression. Cox regression analysis assessed the prognostic value of FOXA1 and Nestin expression.

**Results:**

In breast cancer metastases, FOXA1 expression was associated with Nestin-negativity, GATA3-positivity, ER-positivity, HER2-positivity and non-triple-negative status (*P* < 0.05). In contrast, Nestin expression was associated with FOXA1-negative, GATA3-negative, ER-negative, and triple-negative metastases (*P* < 0.05). Univariate Cox regression analysis showed FOXA1 expression was predictive of overall survival (OS, *P* = 0.00048) and metastasis-free survival (DMFS, *P* = 0.0011), as well as, distant metastasis-free survival in ER-positive patients (*P* = 0.036) and overall survival in ER-negative patients (*P* = 0.024). Multivariate analysis confirmed the significance of FOXA1 for both survival endpoints in metastatic breast cancer patients (OS, *P* = 0.0033; DMFS, *P* = 0.015).

**Conclusions:**

In our study, FOXA1 was expressed mostly in ER-positive breast cancer metastases. Expression of Nestin was related to triple-negative metastases, where brain was the most frequent metastatic site. These findings highlight the clinical utility of FOXA1 and Nestin expression and warrant their inclusion in routine immunohistochemical panels for breast carcinoma.

## Background

Immunohistochemical (IHC) markers for breast cancer (e.g. estrogen and progesterone receptors, Ki67 proliferation marker, and HER2/*neu* testing) are currently used to guide therapeutic decision-making, classify breast cancer subtypes, and act as prognostic and predictive markers [1, 2]. However, these markers are still inadequate in certain subgroups of breast cancer patients. Nevertheless, great efforts have been made to identify novel molecular and IHC markers for clinical management of breast carcinoma that are informative, sensitive, and cost effective.

FOXA1 (forkhead box protein A1) is one of three members in the FOXA related family (forkhead family of transcriptions factors), also known as hepatocyte nuclear factor α (HNF3α). These proteins are often termed “pioneer factors” because of their ability to bind to highly compacted heterochromatin and making genomic regions more accessible to other transcription factors [3, 4]. Namely, FOXA1 can bind to the promotors of more than 100 genes associated with metabolic processes, regulation of the signaling pathways, and the cell cycle [4–6]. In addition, FOXA1 functions as a critical mediator of nuclear steroid receptor signaling via regulation of both androgen and estrogen receptor activity. FOXA1 is expressed in several organs, including the breast, liver, pancreas, urinary bladder, prostate gland, colon and lung. It has a major role in modulating nuclear steroid receptor activity, particularly in cancers of the breast and prostate, and may contribute to pro-tumorigenic phenotypes [7]. FOXA1 is a critical interacting partner of the nuclear hormone receptors, estrogen receptor (ERα) and androgen receptor (AR), which are associated with hormone-regulated cancers such as breast and prostate cancer [3, 8]. FOXA1 is mutated in 1.8% of breast cancers and 3–5% of prostate cancers [9, 10]. Nakshatri et al. reported that a decrease in FOXA1 expression during cancer progression was due to an increase in polycomb complex activity that plays a role in silencing Hox genes through modulation of chromatin structure during embryonic development [11]. The role of E-cadherin is established as an important player in epithelial-mesenchymal-transition (EMT). FOXA1 promotes E-cadherin expression on the protein level by suppressing Slug expression in breast cancer, suggesting that the balance of Foxa1-slug axis regulates EMT-progression [12]. FOXA1 positivity has also been linked with a more favorable prognosis in breast cancer patients treated with Tamoxifen [13, 14]. Reduction in FOXA1 expression contributes to cancer stem cell-like properties in Tamoxifen-resistant breast cancer cells through induction of Interleukin-6 (IL-6) [15]. Horimoto et al. reported that breast cancer patients with high FOXA1 expression tended to develop late recurrences [16].

There are currently few reports on the structure, function and clinical importance of Nestin in breast cancer. Nestin is a type VI intermediate filament protein encoded by the *NES* gene (identified originally as a neural stem cell marker) and participates in cytoskeleton organization [17]. It is expressed in proliferating progenitor cells in embryonic tissues, some adult stem/progenitor cells, and can even be re-expressed in neoplasia [18]. Triple-negative breast cancers have significantly higher *NES* (nestin) mRNA expression than the other breast carcinoma subtypes [19]. Nestin might participate in neovascularization through cytoskeletal changes promoted by the interaction between cancer cells with stem cell properties and endothelial cells lining blood vessels in the tumor stroma. Nowak et al. reported that Nestin expression in endothelial cells lining newly formed breast tumor-associated blood vessels was shown to be associated with the triple-negative subtype, lymph node metastases and shorter overall survival [17, 20]. Nestin contributes to activation of the EMT pathway by regulating the Wnt/β-catenin pathway [21] and may therefore play a role not only in the regulation of mitosis, but in tumor invasiveness [18]. Piras et al. proposed Nestin-positivity in peritumoral stroma, in cells with fibroblast morphology, as evidence of epithelial-stromal interactions [22]. Nestin was significantly associated with angiogenesis and vascular invasion as a sign of early hematogenous spread, but not with lymphatic involvement [23]. In addition, Nestin-positive breast carcinomas lacked *CCND1* and *TOPA2A* gene amplification and occasionally harbored *MYC* gene amplification. There was no correlation between Nestin expression and Topoisomerase IIα expression [18]. Knockdown of Nestin inhibited breast cancer stem cell invasiveness and led to up-regulation of E-cadherin. Simultaneously, mesenchymal markers such as N-cadherin and vimentin were down-regulated [19]. Feng et al. reported that enforced Nestin expression partly counteracted the effect of *SOX10* knockdown on reducing cancer stem cell (CSC) properties.

In this present study, FOXA1 and Nestin expression were examined using immunohistochemistry for 164 breast cancer metastases, followed by Cox regression analysis to assess their prognostic significance in breast cancer.

## Methods

### Patient cohort

We examined 164 breast cancer metastases, corresponding to 162 patients diagnosed between a 10-year period 2004–2014 at Sahlgrenska University Hospital (Gothenburg, Sweden). Consequently, 2/164 patients were diagnosed with two metastases during this time period, including a 65-year-old patient with synchronous bone and brain metastases and a 29-year-old patient with metachronous recurrent brain metastases with a 6-month interval. Nine of the 164 breast cancer metastases were regional axillary lymph node metastases (5.5%), while 155 metastases were distantly located as indicated in Table 1.

**Table 1:** Clinicopathological features for 164 metastatic breast cancer patients

	Total patients (*n* = 164)	FOXA1-positive (*n* = 86)	FOXA1-negative (*n* = 52)	*P*-value	Nestin-positive (*n* = 26)	Nestin-negative (*n* = 114)	*P*-value
Age (y)				0.21			0.29
≤ 55	59 (36%)	22 (26%)	20 (38%)		10 (38%)	33 (29%)	
56–80	94 (57%)	59 (69%)	28 (33%)		16 (62%)	72 (63%)	
> 80	11 (7%)	5 (6%)	4 (5%)		0 (0%)	9 (8%)	
Metastasic site				0.31			0.06
Abdomen/GI tract/Liver	44 (27%)	29 (34%)	9 (17%)		5 (19%)	34 (30%)	
Axillary lymph node/sentinel node	11 (7%)	5 (6%)	3 (6%)		1 (4%)	7 (6%)	
Brain	29 (18%)	7 (8%)	9 (17%)		8 (31%)	9 (8%)	
Cervical lymph nodes	3 (2%)	2 (2%)	1 (2%)		0 (0%)	3 (3%)	
Gynecological site	9 (5%)	2 (2%)	7 (13%)		2 (8%)	7 (6%)	
Skeleton	37 (23%)	20 (23%)	15 (29%)		3 (12%)	32 (28%)	
Skin	11 (7%)	7 (8%)	4 (8%)		3 (12%)	8 (7%)	
Thorax/Lung	20 (12%)	14 (16%)	4 (8%)		4 (15%)	14 (12%)	
Not available	0 (0%)	0 (0%)	0 (0%)		0 (0%)	0 (0%)	
GATA3 status				**2.80E-04**			**0.039**
Positive	154 (94%)	86 (100%)	44 (85%)		22 (85%)	110 (96%)	
Negative	10 (6%)	0 (0%)	8 (15%)		4 (15%)	4 (4%)	
Not available	0 (0%)	0 (0%)	0 (0%)		0 (0%)	0 (0%)	
Mammoglobin status				0.080			0.83
Positive	84 (51%)	47 (55%)	20 (38%)		12 (46%)	57 (50%)	
Negative	80 (49%)	39 (45%)	32 (62%)		14 (54%)	57 (50%)	
Not available	0 (0%)	0 (0%)	0 (0%)		0 (0%)	0 (0%)	
FOXA1 status							**0.0039**
Positive	86 (52%)	–	–		9 (35%)	77 (68%)	
Negative	52 (32%)	–	–		17 (65%)	35 (31%)	
Not available	26 (16%)	–	–		0 (0%)	2 (2%)	
Nestin status				**0.0017**			
Positive	26 (16%)	9 (10%)	17 (33%)		–	–	
Negative	114 (70%)	77 (90%)	35 (67%)		–	–	
Not available	24 (15%)	0 (0%)	0 (0%)		–	–	
Primary tumor differentiation				0.29			0.96
Well	10 (6%)	5 (6%)	3 (6%)		1 (4%)	7 (6%)	
Moderate	49 (30%)	31 (36%)	11 (21%)		7 (27%)	36 (32%)	
Poor	59 (36%)	27 (31%)	19 (37%)		9 (35%)	37 (32%)	
Not available	46 (28%)	23 (27%)	19 (37%)		9 (35%)	34 (30%)	
Axillary lymph node status				**0.047**			1.00
pN0	55 (34%)	34 (40%)	11 (21%)		9 (35%)	37 (32%)	
pN1	78 (48%)	38 (44%)	26 (50%)		12 (46%)	53 (46%)	
Not available	31 (19%)	14 (16%)	15 (29%)		5 (19%)	24 (21%)	
ER status				**2.09E-05**			**0.024**
Positive	115 (70%)	74 (86%)	27 (52%)		14 (54%)	89 (78%)	
Negative	49 (30%)	12 (14%)	25 (48%)		12 (46%)	25 (22%)	
Not available	0 (0%)	0 (0%)	0 (0%)		0 (0%)	0 (0%)	
PR status				0.07			0.50
Positive	57 (35%)	39 (45%)	15 (29%)		8 (31%)	46 (40%)	
Negative	107 (65%)	47 (55%)	37 (71%)		18 (69%)	68 (60%)	
Not available	0 (0%)	0 (0%)	0 (0%)		0 (0%)	0 (0%)	
HER2/neu status				**0.0031**			0.42
Positive	45 (27%)	29 (34%)	6 (12%)		4 (15%)	31 (27%)	
Negative	116 (71%)	55 (64%)	46 (88%)		22 (85%)	81 (71%)	
Not available	3 (2%)	2 (2%)	0 (0%)		0 (0%)	2 (2%)	
Triple negative status				**1.92E-06**			**0.0021**
Triple negative	27 (16%)	4 (5%)	19 (37%)		10 (38%)	13 (11%)	
Non-triple negative	137 (84%)	82 (95%)	33 (63%)		16 (62%)	101 (89%)	
Not available	0 (0%)	0 (0%)	0 (0%)		0 (0%)	0 (0%)	
Overall survival				0.17			1.00
Deceased	88 (54%)	68 (79%)	46 (88%)		22 (85%)	94 (82%)	
Survivor	76 (46%)	18 (21%)	6 (12%)		4 (15%)	20 (18%)	

NOTE: *P*-values were calculated using the Fisher's exact test (FOXA1-positive vs. FOXA1-negative or Nestin-positive vs Nestin-negative. Statistically significant variables (*p* <0.05) are displayed in bold text

In total, 20/162 patients (12.3%) were still alive at the start of the study (henceforth termed long-term survivors). For long-term survivors, the interval between the primary breast cancer and metastases varied between 2 and 35 years (average 8.25 years). However, three of the 20 patients developed metastases within 12 months. The youngest long-term survivor was 44-years-old at the time of metastasis, while the oldest patient was 82-years-old (average age was 58.6 years).

In addition, 11 of the 162 patients (6.8%) had already been diagnosed with metastatic breast cancer at the time of diagnosis of the primary tumor. Of these, 3/11 patients had already developed axillary lymph node metastases at the time of diagnosis, 3/11 patients had bone metastases, 1/11 patient had a liver metastasis, 2/11 patients had metastases in the abdomen and peritoneum, 1/11 patient was diagnosed with breast cancer metastases in the ovarium, and 1/11 patient presented with breast cancer metastases in the soft tissue in the parasternal region.

### Evaluation of immunohistochemistry (IHC)

Full-face formalin-fixed paraffin-embedded (FFPE) specimens for the 164 breast cancer metastases were obtained from the Department of Clinical Pathology at Sahlgrenska University Hospital in accordance with the Declaration of Helsinki and approved by the Medical Faculty Research Ethics Committee (Gothenburg, Sweden). Each FFPE specimen was examined for mammaglobin, ER/PR, cytokeratin 7 (CK7), cytokeratin 20 (CK20), and HercepTest at the time of diagnosis and retrospectively analyzed by immunohistochemistry (IHC) for GATA3, FOXA1 and Nestin expression. Four micrometer FFPE (formalin fixed paraffin embedded) sections were pretreated using the Dako PTLink system (Dako, Carpinteria, CA) and processed on an automated DAKO Autostainer platform using the Dako Envision™ FLEX High pH Link Kit (pH 9; Table 2). Peroxidase-catalyzed diaminobenzidine was used as the chromogen, followed by hematoxylin counterstain. Normal breast gland tissue and tonsillar tissue were used as positive controls for FOXA1 and Nestin, respectively. A Leica DM4000 B microscope was used in the experiments. Immunostaining was evaluated by a breast pathologist, blinded to patient clinical outcome. For each metastasis, the pattern of nuclear FOXA1, nuclear GATA3, and cytoplasmic Nestin staining was recorded together with the extent (% of positively stained tumor cell nuclei or tumor cell cytoplasm using deciles: 0%; 5, 10, 40, 90%, etc.). Positivity thresholds were set at ≥1%. However, < 20% was rarely noticed among positive cases. An overall assessment of IHC was given, not focusing on hot spots.

**Table 2:** Summary of immunohistochemical stains and antigen retrieval techniques

Antibody	Manufacturer	Clone	Dilution	Antigen retrieval
GATA3	CELL MARQUE 390M-16	L50–823	1:200	TRS high, pH 9.0
FOXA1	CELL MARQUE 405M-16	2F83	1:100	TRS high, pH 9.0
Nestin	CELL MARQUE 388M-16	10C2	1:50	TRS high, pH 9.0

### Statistical analysis

The statistical analyses were performed using a 0.05 *P*-value cutoff in R/Bioconductor (version 3.3.2) and all *P*-values are two-sided. The relationship between clinicopathological features and FOXA1 and Nestin protein expression patterns was evaluated using two-tailed Fisher’s exact test (R stats package). Univariate Cox proportional hazard models were calculated for FOXA1 and Nestin expression using overall survival (OS) and distant metastasis-free survival (DMFS; survival (v2.43–1)). Multivariate analysis was conducted using the Cox proportional hazard model for OS and DMFS with FOXA1 and Nestin expression after adjusting for clinicopathological features (age at diagnosis, metastatic site, Mammoglobin status, GATA3 status, histological grade, axillary lymph node status, ER/PgR status, HER2/neu status, and triple-negative status). Breast cancer survival rates were defined as a) time from initial diagnosis of the primary breast carcinoma to death from any cause for OS and b) time from initial diagnosis of the primary breast carcinoma to distant metastasis for DMFS. Survival rates were depicted with Kaplan–Meier curves and tested with log-rank test (survminer (v0.4.3)). Forest plots were generated using the forestplot package (v1.7.2).

## Results

### Association between clinicopathological features and FOXA1 and nestin expression

**Fig. 1:**
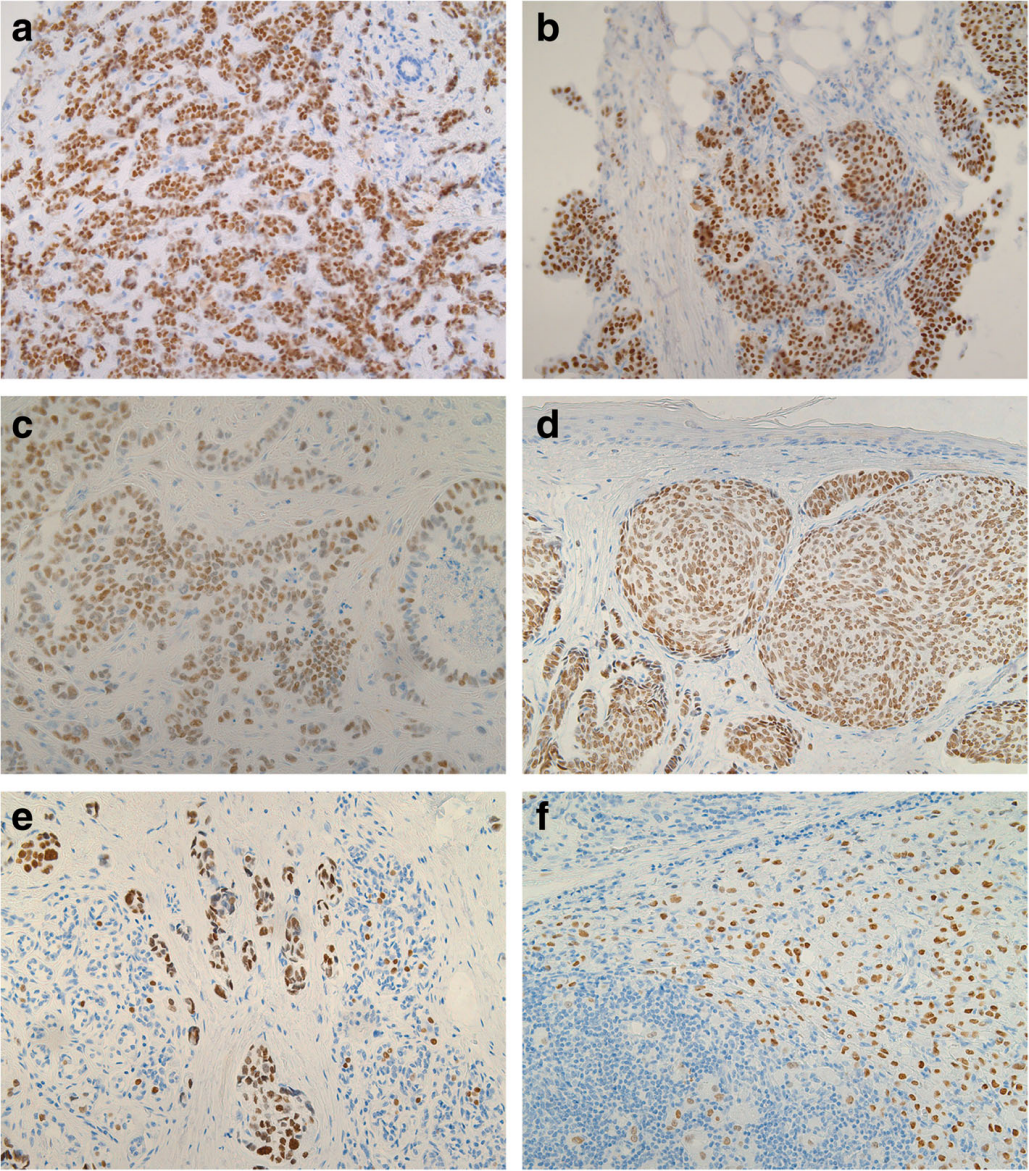
Positive FOXA1 immunostaining in breast cancer metastases, including **a** liver, **b** mediastinum, **c** cerebellum, **d** skin, **e** breast with recurrent cancer, and **f** axillary lymph node (100 x magnification each)

**Fig. 2:**
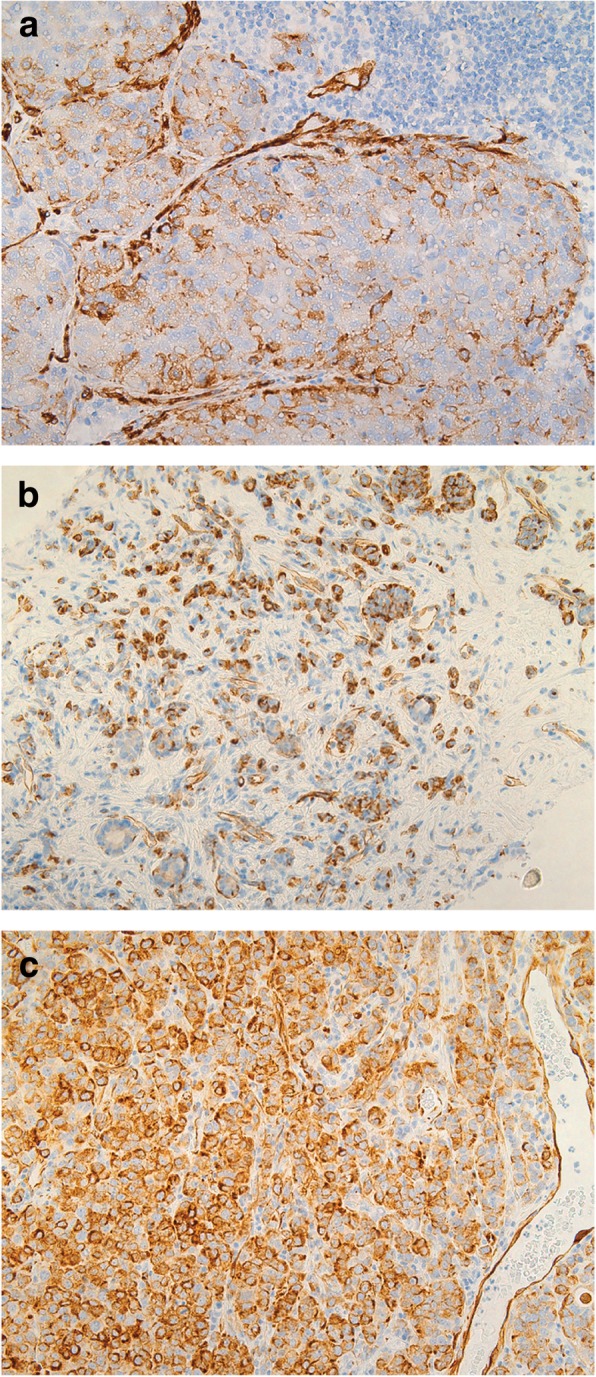
Positive Nestin immunostaining in breast cancer metastases, including **a** sentinel node (200 x magnification), **b** liver “(100 x magnification)”, and **c** cerebellum (200 x magnification)

Immunohistochemical analysis of FOXA1 and Nestin expression in breast cancer metastases revealed FOXA1-positivity and Nestin-positivity in 52 and 16% of the metastatic samples, respectively (Fig. 1, 2 and Table 1). A strong association between FOXA1 expression and GATA3-positivity (*P* < 0.001) was found, with FOXA1 expression detected in 100% of GATA3-positive cases. In addition, FOXA1 expression was found in patients with no spread to the axillary lymph nodes and tumors characterized as Nestin-negative, ER-positive, HER2-positive, and non triple-negative (*P* < 0.05). In contrast, Nestin-positivity was inversely associated with GATA3, FOXA1, and ER expression, and triple negative status (*P* < 0.05). Hence, protein expression for FOXA1 and Nestin were inversely related. No association was found between FOXA1 and Nestin expression and other clinicopathological features, such as metastatic site, age, mammoglobin status, primary tumor differentiation, axillary lymph node status, progesterone receptor status or overall survival.

### Prognostic significance of FOXA1 and nestin expression in breast cancer metastases

**Fig. 3:**
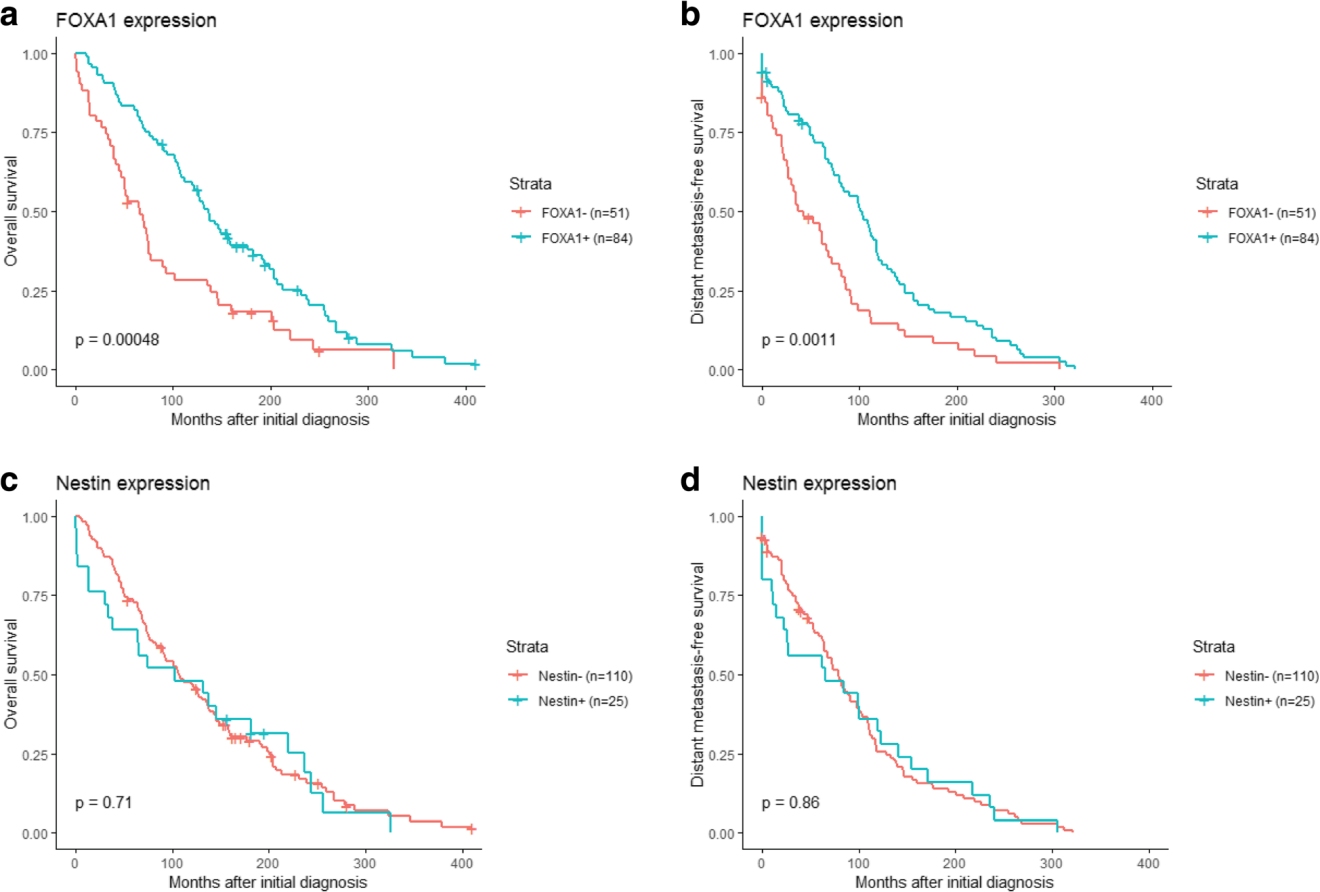
Kaplan–Meier analysis of FOXA1 and Nestin expression in metastatic breast cancer patients. **a** and **c** Estimates of the probability of overall survival (OS) in the patient cohort. *P*-values were calculated using the log-rank test and Cox proportional hazards regression. **b** and **d** Estimates of the probability of distant metastasis-free survival (DMFS) in the patient cohort. *P*-values were calculated using the log-rank test and Cox proportional hazards regression. The x-axes depict months after initial diagnosis and the y-axes depict survival rates

**Fig. 4:**
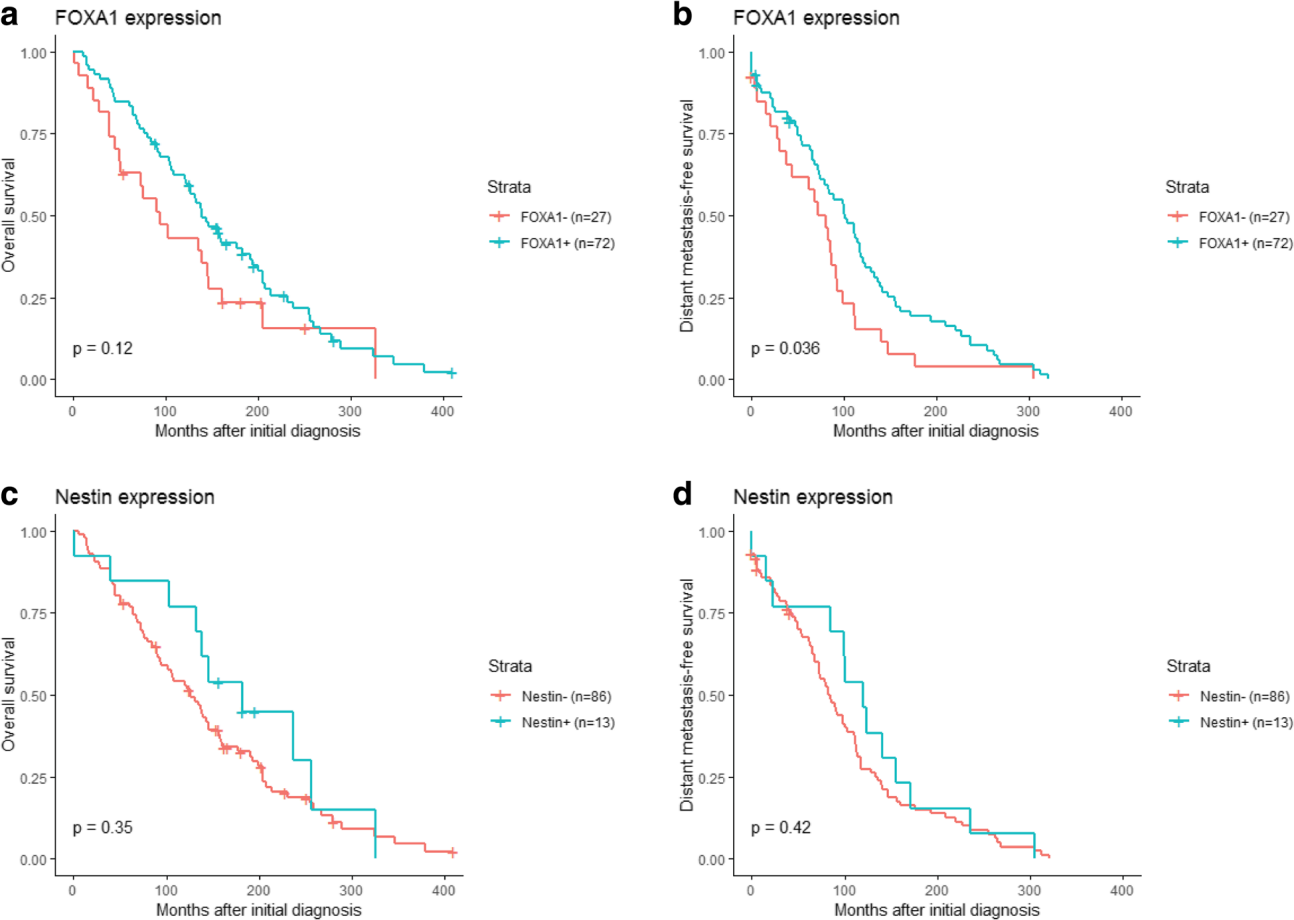
Kaplan–Meier analysis of FOXA1 and Nestin expression in ER-positive, metastatic breast cancer patients. **a** and **c** Estimates of the probability of overall survival (OS) in the patient cohort. *P*-values were calculated using the log-rank test and Cox proportional hazards regression. **b** and **d** Estimates of the probability of distant metastasis-free survival (DMFS) in the patient cohort. *P*-values were calculated using the log-rank test and Cox proportional hazards regression. The x-axes depict months after initial diagnosis and the y-axes depict survival rates

**Fig. 5:**
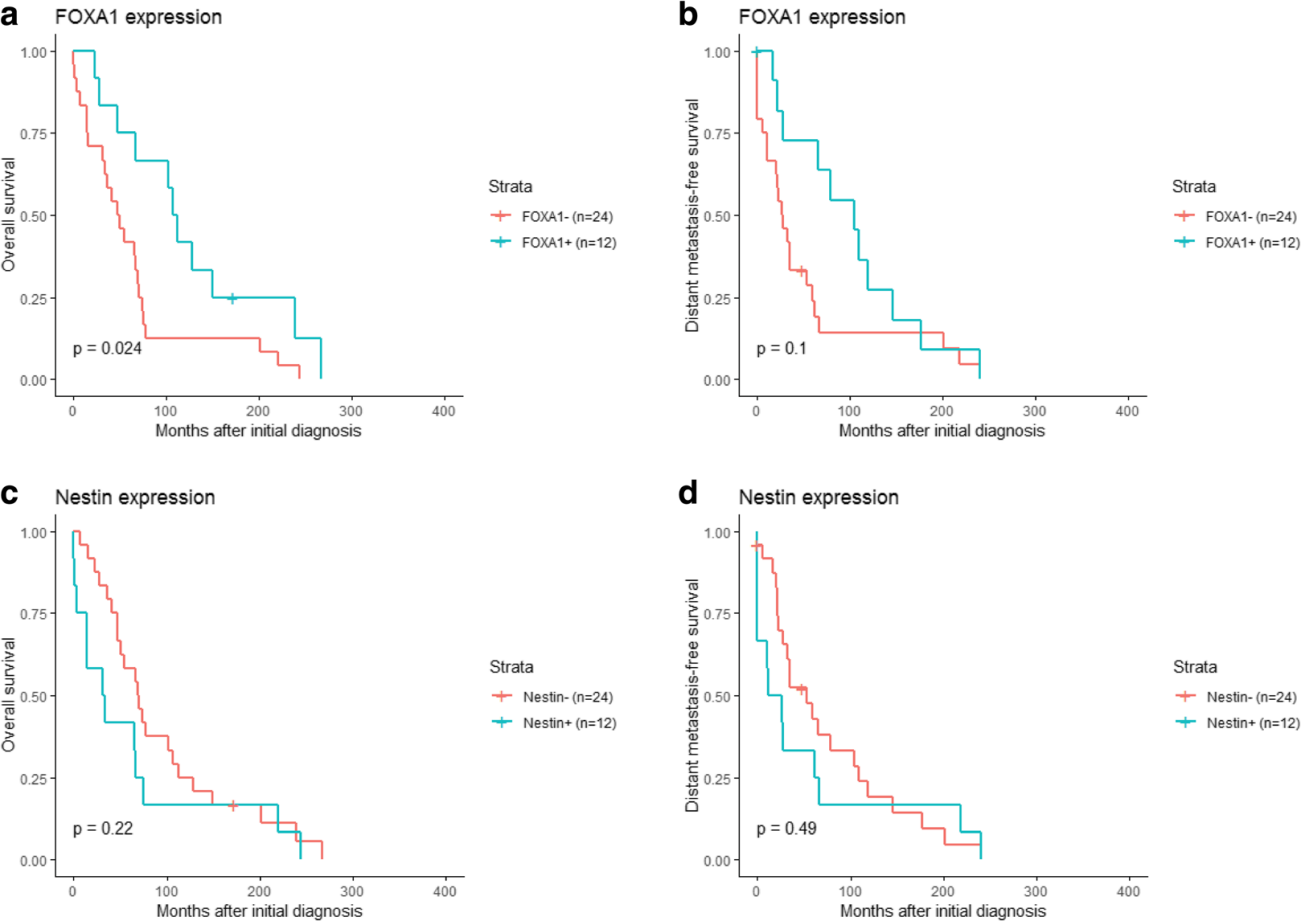
Kaplan–Meier analysis of FOXA1 and Nestin expression in ER-negative, metastatic breast cancer patients. **a** and **c** Estimates of the probability of overall survival (OS) in the patient cohort. *P*-values were calculated using the log-rank test and Cox proportional hazards regression. **b** and **d** Estimates of the probability of distant metastasis-free survival (DMFS) in the patient cohort. *P*-values were calculated using the log-rank test and Cox proportional hazards regression. The x-axes depict months after initial diagnosis and the y-axes depict survival rates

**Fig. 6:**
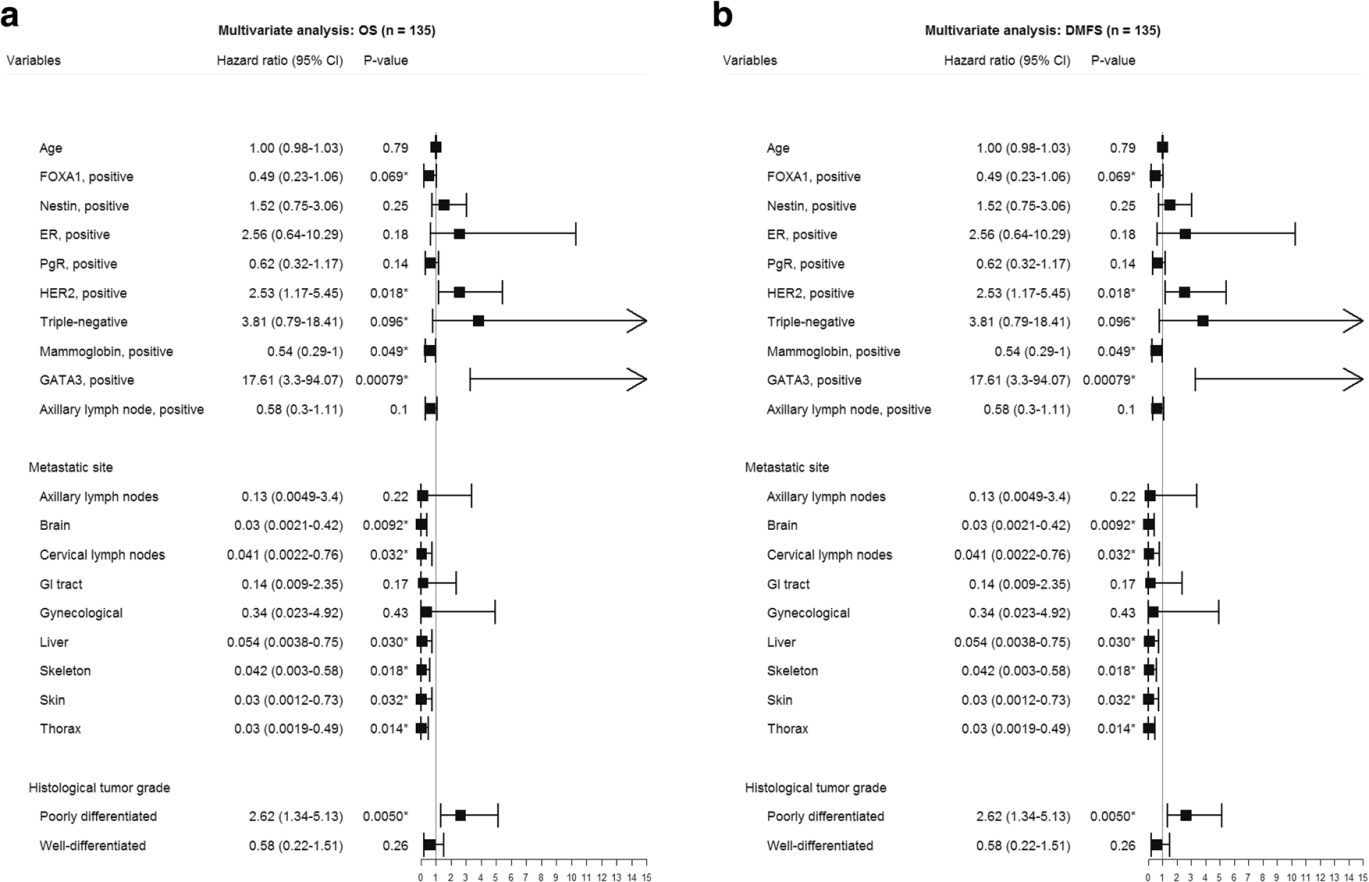
Forest plots illustrating multivariate Cox regression analysis of the prognostic impact of FOXA1 and Nestin expression on, **a** overall survival (OS), and **b** distant metastasis-free survival (DMFS) in metastatic breast cancer patients

Univariate Cox regression analysis showed an association between FOXA1 expression and both overall survival (OS; *P* = 0.00048) and distant metastasis-free survival (DMFS; *P* = 0.0011; Fig. 3). In contrast, Nestin expression was not associated with clinical outcome in the patient cohort (OS and DMFS; *P* > 0.05). Stratification of the patient cohort by ER status revealed that FOXA1 expression was a predictor of DMFS in ER-positive patients (*P* = 0.036) and OS in ER-negative patients (*P* = 0.024). No significant correlation was found between Nestin expression and either survival endpoint in patients stratified by ER status (Fig. 4, 5). Multivariate analysis using a Cox proportional hazards model was performed to assess the prognostic value of FOXA1 and Nestin expression after adjusting for other clinicopathological features. Interestingly, multivariate analysis confirmed the clinical relevance of FOXA1 expression and survival in metastatic breast cancer patients (OS, *P* = 0.010; DMFS, *P* = 0.069; Fig. 6). In addition, metastatic site (i.e. brain, cervical lymph nodes, GI tract, gynecological, liver, skeleton, skin, thorax), mammoglobin-positivity, and GATA3-positivity were associated with OS (*P* < 0.05). For DMFS, metastatic site (i.e. brain, cervical lymph nodes, liver, skeleton, skin, thorax), mammoglobin-positivity, GATA3-positivity, HER2-positivity, triple-negative status, and high histological grade were statistically significant (*P* < 0.05).

## Discussion

To date, immunohistochemical analysis of FOXA1 and Nestin expression has only been performed using primary breast carcinomas. In the present study, only the breast cancer metastases were available, all of which had been diagnosed at Sahlgrenska University Hospital (Gothenburg, Sweden). Unfortunately, the FFPE samples for the primary breast carcinomas were more difficult to retrieve and the patients had been diagnosed and operated for the primary tumors at different hospitals in Western Sweden. It was therefore not possible to evaluate FOXA1 and Nestin expression in the primary tumor at this time. In future studies, it would be interesting to investigate FOXA1 and Nestin expression in the primary breast carcinoma corresponding to the 164 metastatic breast carcinomas investigated here. This is the first report, to our knowledge, showing FOXA1 and Nestin expression in breast cancer metastases.

Previous studies have shown that FOXA1 expression was inversely associated with tumor size, histological grade, lymph node status, HER2 expression and lymph vascular invasion, while GATA3 expression showed an inversed association with tumor histological grade and HER2 status [24, 25]. Both FOXA1 and GATA3 were associated with ERα and progesterone positivity. Over 80% of FOXA1 and GATA3-positive breast carcinomas belonged to the luminal A subtype. FOXA1 have been shown to have a dual role, either as a growth stimulator or a repressor. It functions as a tumor promotor in initial stages, but as a tumor repressor in the later stages [4, 5]. Cross talk between FOXA1 and ER can favor the expression of differentiation-associated genes, and not the proliferation-associated genes that results in well differentiated breast carcinomas with estrogen receptor positivity, which per se indicates a good prognosis [5] [26]. As a tumor suppressor, FOXA1 overexpression might block metastatic progression by influencing the expression of *p27* (BRCA1 associated cell cycle inhibitor) and promoting E-Cadherin expression [4].

According to the statistical analysis, our results correlated well with the data reported in the literature. Breast cancer patients with tumors expressing FOXA1 may have a better clinical outcome because FOXA1 and GATA3 are expressed in close association with ERα (Estrogen receptor α), by encoding for transcription factors and have a potential involvement in ERα-mediated breast cancer development [24]. Because FOXA1 was clinically relevant in several studies, its practical application in routine pathology might be advisable. Ademuyiwa et al. suggested that FOXA1 immunostaining could function as a more cost effective pathological marker than the Oncotype DX multigene prognosis assay, because FOXA1 negatively correlated with the recurrence score [27]. FOXA1 is already included in PAM50, which is also a gene profiling kit for breast cancer patients. A decade has passed since Thorat et al. suggested FOXA1 be used in treatment decision making, as it showed a prognostic ability in low-risk breast cancer [26]. Moreover, neoadjuvant chemotherapy (NAC) could be recommended for breast cancer patients with low FOXA1 expression [16]. FOXA1 expression was also been evaluated after NAC, which was significantly associated with distant disease-free survival, both by univariate and multivariate analyses [28].

FOXA1 expression has also been assessed in primary carcinomas from other anatomic sites than breast carcinoma. Interestingly, *FOXA1* DNA amplification was only observed in metastatic prostate cancer samples, which was associated with increased proliferation and tumor size [10, 29]. In breast cancer, *FOXA1* amplification was a marker of favorable prognosis [3, 13]. Molecular classification of breast cancer is a helpful tool with respect to patient clinical outcome. Robinson et al. reported that the majority of breast cancers expressing FOXA1 were mainly of Luminal A type (5/8), Luminal B type (2/8), and HER2 positive subtype (1/8). Robinson et al. also reported that breast cancer patients with *FOXA1* amplification have a much better prognosis and treatment response [3]. Decreased FOXA1 levels in pleural metastases was associated with endocrine therapy resistance [30].

While FOXA1 and GATA3 are significant predictors of favorable outcome in breast carcinoma (longer disease-free interval and overall survival), Nestin expression is associated with a more unfavorable prognosis [13, 24, 31]. Our results correlated well with these findings, as FOXA1 expression was associated with both better OS and DMFS. According to our data, Nestin was not associated with OS and DMFS, but was correlated with triple-negative status which is generally connected with poor patient outcome. Moreover, Nestin might become a potential treatment target because knockdown of Nestin demonstrated reduced cell motility in several different carcinomas, including prostate, colorectal-, nasopharyngeal- and lung carcinoma [23]. Here, we show FOXA1 expression in breast cancer metastases, but it is still unclear whether other FOXA family members such as FOXA2 and FOXA3 also play a role in breast cancer.

## Conclusions

In the present study, FOXA1 and Nestin expression in breast cancer metastases was associated with specific breast cancer subtypes (luminal phenotype versus triple-negative breast cancer metastases). GATA3 has been already used as a specific marker to identify breast cancer metastases [32]. We propose the inclusion of FOXA1 and Nestin immunohistochemical evaluation in current immune panels for breast carcinoma to improve prognostication and therapy choice.

## Abbreviations

AR: androgen receptor
CK 20: cytokeratin 20
CK 7: cytokeratin 7
CSC: cancer stem cell
DMFS: distant metastasis-free survival
EMT: epithelial-mesenchymal-transition
ER: estrogen receptor
ERα: estrogen receptor α
FFPE: full-face formalin-fixed paraffin-embedded
FOXA1: forkhead box protein A1
GI-tract: gastrointestinal tract
HNF3α: hepatocyte nuclear factor α
IHC: immunohistochemistry
NAC: neoadjuvant chemotherapy
OS: overall survival
